# *In situ* polymerisation of isoeugenol as a green consolidation method for waterlogged archaeological wood

**DOI:** 10.1038/srep46481

**Published:** 2017-04-27

**Authors:** Emily McHale, Calin C. Steindal, Hartmut Kutzke, Tore Benneche, Stephen E. Harding

**Affiliations:** 1 Museum of Cultural History, University of Oslo, Postbox 6762 St. Olavs plass, 0130 Oslo, Norway; 2 Department of Chemistry, University of Oslo, Postbox 1033 Blindern, 0315 Oslo, Norway; 3 National Centre for Macromolecular Hydrodynamics, University of Nottingham, Sutton Bonington, LE12 5RD, United Kingdom

## Abstract

Waterlogged archaeological wood is often in need of consolidation prior to drying to prevent shrinkage and cracking of the object. There is a need for new greener materials (than for example polyethylene glycol) and methods for consolidation to be developed. The use of wood-based components could provide good interaction between the consolidant and the remaining wood structure and would also support a shift away from fossil fuel-based materials to those with more sustainable sources. Based on this, lignin-like structures have been investigated for their ability to consolidate waterlogged archaeological wood. The *in situ* formation of a lignin-like material has been carried out using isoeugenol polymerised by horse radish peroxidase in aqueous solution. The formation of the oligomeric/polymeric materials within the wood following this reaction has been determined by Attenuated Total Reflectance Fourier Transform Infra Red (ATR-FTIR) spectroscopy. The oligomers remaining in solution have been characterised by ATR-FTIR and nuclear magnetic resonance (NMR) spectroscopy as well as analytical ultracentrifugation, showing that they have a weight average M_w_ of 0.4–0.9 kDa and a lignin-like structure rich in the β-5′ moiety. Therefore, this approach is proposed as a basis to further develop a green consolidation method for waterlogged archaeological wood.

Archaeological wood provides an important source of information about previous cultures and wooden finds^1^, such as shipwrecks, which are often popular museum attractions. However, when compared to other materials, archaeological wood is rarely found due to its biodegradable nature^2^,^3^. The level of degradation seen does not only depend on the age of the object but also on the environment in which it has survived and the species of the wood^4^,^5^. The most common environments from which archaeological wood is excavated are wet environments such as on the sea bed or in bogs^1^,^2^. In such environments the transport of oxygen is limited and, thus, the rapid degradation of the cell wall by fungi is also limited^6^. Instead a slow degradation of the polysaccharides by bacteria takes place. As the polysaccharides are depleted, water is absorbed into the cell wall, potentially causing swelling^5^. Waterlogged archaeological wood will often distort and shrink if dried without treatment^4^. The shrinkage occurs in the cell wall and occurs below the fibre saturation point because of desorption^2^,^7^. Further damage to the object from drying can come in the form of cell wall collapse, wherein the cell walls fold over each other as a result of capillary drying tension above the fibre saturation point^1^.

As a result, waterlogged archaeological wood often needs to be impregnated with a consolidant before drying^3^,^8^. The consolidant provides mechanical strength to the cell walls and prevents, or limits, shrinkage and collapse. It is important that the consolidant is compatible with the remaining wood structure and is, ideally, able to interact to offer further support. Currently the most widely used consolidant is polyethylene glycol (PEG) of suitably chosen molecular weight and polydispersity. PEG is produced from fossil fuel-based sources under either acidic or basic conditions. Often, ethylene oxide is used as the monomer, which is extremely flammable and toxic. PEG itself, however, is not toxic and is water soluble, but as part of the consolidation treatment a large quantity of PEG that has not impregnated the wood, is wasted. It is also not a natural biological polymer. As a consequence there has been an increased interest over the past decade in green materials for consolidation^3^,^9^,^10^,^11^,^12^,^13^,^14^,^15^,^16^, such as the non-reducing sugars lactose and trehalose^10^,^17^,^18^. However, treatment with sugars requires the use of biocides and this poses both environmental and health concerns^1^. Therefore, new sustainably sourced consolidation systems that do not require the use of biocides are needed.

It has been proposed that, due to the high chemical and biological resistance of lignin, a lignin-based material could provide a new green system for consolidation. Lignin is often the major component remaining within archaeological wood and, as such, is the material with which compatibility and interaction is needed^1^,^4^,^19^,^20^. Lignin is also currently wasted in tonnes every year as a by-product of the pulp and paper industry. This makes it a potential sustainable source for a consolidant^21^,^22^. Lignin, however, is not soluble in water and has limited solubility in most commonly used organic solvents^23^,^24^. Therefore, our group has previously reported preliminary work into the use of synthetic lignin-like oligomers (dehydrogenated polymers, DHPs) for the consolidation of waterlogged archaeological wood^25^,^26^.

Traditionally lignin-like oligomers have been synthesised by peroxidases and laccases from coniferyl alcohol^27^,^28^. However, coniferyl alcohol is not readily available and needs to be synthesised from ferrulic acid. *Isoeugenol,* on the other hand, is structurally very similar to coniferyl alcohol, Fig. 1a, and is readily available and relatively cheap. It can be sourced from the essential oil of the Asian tree ylang-ylang (*Cananga odorata*), has very low toxicity and is often used as a flavour and fragrance additive. Its low cost makes it more attractive than coniferyl alcohol for the cultural heritage sector where funds are often limited.

Synthetic lignin oligomers are only very poorly water-soluble^29^ and organic solvents are needed for their impregnation into wood^25^. In order to reduce the use of organic solvents, *in situ* polymerisation of the lignin-like consolidant was investigated. The initial aim was to synthesise lignin-like oligomers that were rich in the β-5′ moiety and low in the β-O-4′ moiety, Fig. 1b, as the latter is most readily cleaved in lignin^30^,^31^. The β-5′ moiety, by contrast, is less readily cleaved and therefore is more favourable as a material for long-term conservation.

Orlandi *et al*. have previously carried out research into the *in situ* polymerisation of isoeugenol with a bulky copper salen catalyst in waterlogged archaeological wood^32^,^33^. However, it was found that the maximum moisture content (MMC %) of the wood treated in this way was reduced, suggesting some level of consolidation had occurred. The polymer formed was found to have a higher proportion of the β-O-4′ moiety than deemed desirable for this investigation. Based on the literature, horse radish peroxidase (HRP) was proposed as an alternative catalyst as it promotes the β-5′ moiety, and thus would result in a more stable oligomer or polymer^34^. Therefore, this paper investigates the potential for isoeugenol to be polymerised *in situ* using HRP. At this time the consolidation ability of the material formed was not investigated as the aim was to determine the potential for *in situ* polymerisation using this approach.

## Results and Discussion

**Figure 1:**
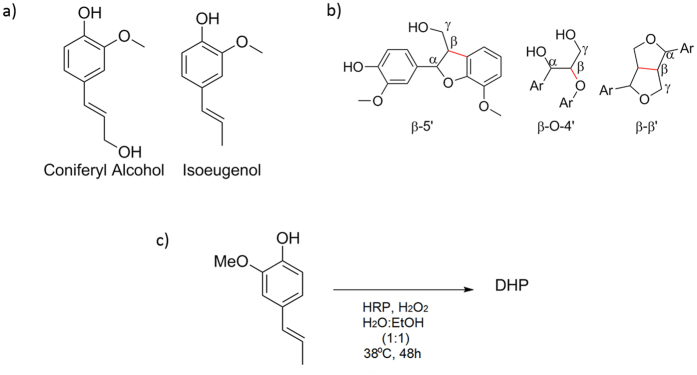
Isoeugenol and lignin (**a**) The chemical structures of coniferyl alcohol and isoeugenol. (**b**) The structure of the major interconnecting units of lignin monomers with the bonds giving their names shown in red. (**c**) Reaction scheme for the polymerisation of isoeugenol with HRP.

**Figure 2:**
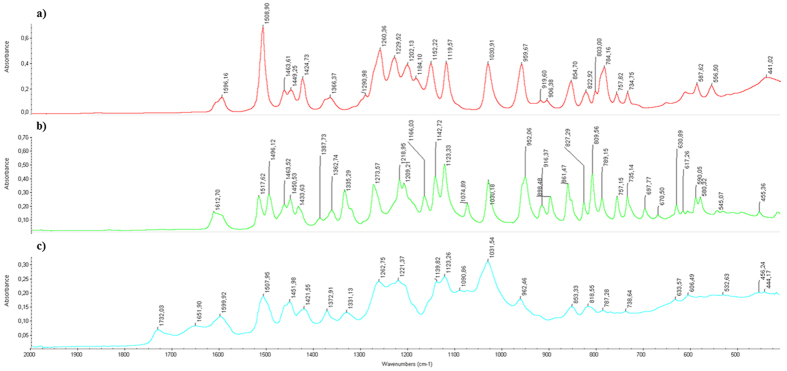
ATR-FTIR spectra of (**a**) isoeugenol, (**b**) di-isoeugenol (β-5′) and (**c**) polymerisation product of isoeugenol. A significant difference can be seen between spectra **a, b** and **c** with the emergence of a bands at 1651 and 1732 cm^−1^ indicating that polymerisation had occurred.

In order to determine the activity of HRP towards isoeugenol, the polymerisation was first carried out in the absence of archaeological wood, Fig. 1c. Polymerisation of isoeugenol was confirmed by FTIR spectroscopy, Fig. 2. Comparison between the polymerisation product, isoeugenol and di-isoeugenol showed that polymerisation had occurred and not simply dimerization. Most notable were the emergence of bands at 1732, 1651 and 1331 cm^−1^ which are assigned to an aldehyde at the terminal end of the oligomers, a conjugated alkene and a C-O stretch respectively.

**Figure 3:**
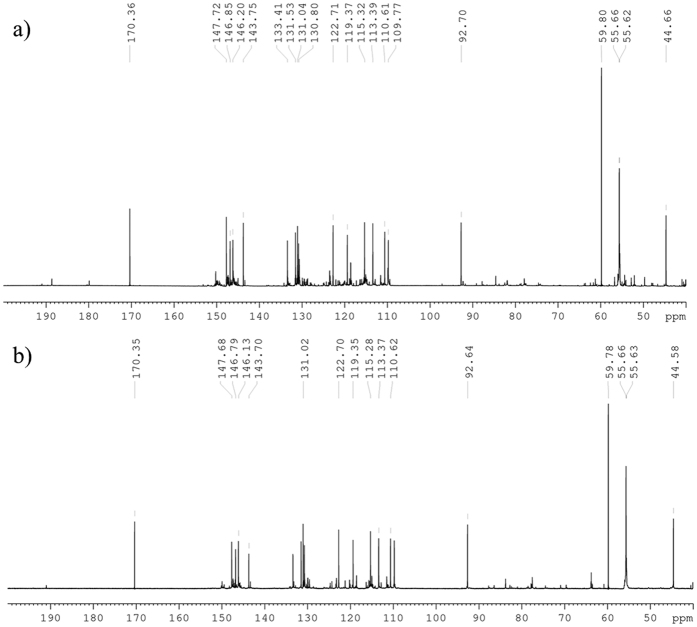
^^13^^C NMR spectra of (**a**) isoeugenol polymerised with HRP and (**b**) isoeugenol polymerised with HRP in the presence of archaeological wood, **IE1**. The distribution of peaks is almost identical for both. The peak at 92.70 ppm refers to the α-C in the β-5′ moiety.

The ^13^C NMR spectrum, Fig. 3, showed typical features of a lignin-like structure. The peak at 92.7 ppm refers to the α-C of the β-5′ moiety, which has a significantly higher intensity than the peaks at 85.8 and 81.9 ppm which are due to β-C of the β–β′ and β-O-4′ respectively. Although the β-5′ moiety was expected as the predominant linkage it was not expected that such a high proportion would be formed. Only trace amounts of the β-O-4′ and β–β′ moieties could be detected by NMR spectroscopy. At neutral pH it is common with coniferyl alcohol that the β-5′ moiety is predominant; however, there is usually a significant contribution from β-O-4′ and β–β′ moieties^34^,^35^. It is proposed that the reactivity of isoeugenol towards the formation of the different interconnecting moieties would be similar if not the same. There was an additional peak at 190.1 ppm, that has been assigned to a oligomer chain terminal aldehyde formed by oxidation on the C3 chain, which had also been observed during polymerisation of isoeugenol with a bulky copper salen catalyst^25^. This showed that the polymerisation of isoeugenol with HRP was promising and as such it was applied to *in situ* polymerisation.

The first step of the *in situ* polymerisation was to impregnate the waterlogged archaeological wood (620% MMC, density of 0.146 g/mL determined to be in a medium state of degradation) with the isoeugenol monomer via bulk addition. Bulk addition was proposed to favour impregnation compared to dropwise addition of the monomer as it created a larger concentration gradient between the inside of the wood and the bulk solution. This was carried out for either two, four or seven days as shown in Table 1. As wood, and even more so archaeological wood, is very unhomogeneous, it was not clear how fast diffusion into the wood would proceed. Unfortunately, due to the overlapping nature of the FTIR bands of isoeugenol and wood, it was not possible to track the isoeugenol impregnation over time.

The second step was to add HRP and H_2_O_2_ to the solution to initiate polymerisation. HRP was proposed to be too large a molecule to efficiently penetrate the wood and therefore pre-impregnation of HRP was not investigated. When considering the addition rate of H_2_O_2_ at this stage it was decided to use bulk addition in order to be certain that any changes in the formed oligomers between the reaction lengths and temperatures were not a result of variations in addition rate. As a previous study had shown that lignin-like oligomers were able to offer mechanical support to waterlogged archaeological wood in a similar state of degradation it was proposed that for this investigation the focus would be to synthesise oligomers *in situ*^25^,^26^. However, it is proposed that a further study would be carried out to optimise the addition rate of H_2_O_2_ in order to get a polymer with as high a molecular weight as possible for consolidation.

**Figure 4:**
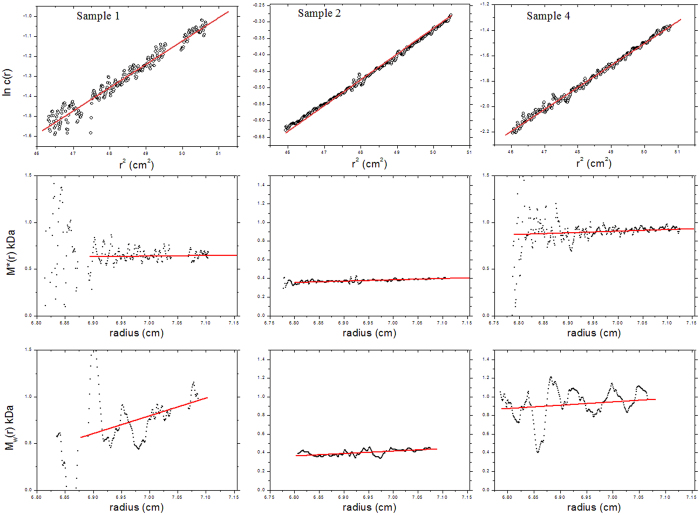
Sedimentation equilibrium in the AUC. The top row of plots show ln c(r) vs r^2^ for IE1, IE2 and IE4. As the fit lines are linear, it can be assumed that the samples are all near-monodisperse. The second row of plots shows M*(r) vs r, the plots have been extrapolated to the cell base (radius = 7.155 cm) to give the weight average molar mass over the whole distribution (M_w_) (Creeth and Harding 1982). The bottom set of plots show local or point average molar masses M_w_(r) at different positions r by taking the local gradients along the ln c(r) vs r^2^ curve. These plots are noisier than the estimate for the whole distribution M_w_ because of the differentiations of the data.

FTIR spectroscopy was used to confirm that polymerisation had occurred in the solutions of all samples (Supporting Information). Comparison with the FTIR spectrum from the reference reaction (Fig. 1c) and isoeugenol (Fig. 1a) allowed a quick screening for the occurrence of polymerisation in solution. ^13^C NMR spectroscopic analysis of **IE1** (Fig. 3b) from the solution confirmed polymerisation, giving a very similar spectrum to that of the reference material (Fig. 3a). This showed that, as with the reference material, the oligomers which formed in solution were rich in the β-5′ moiety, containing only trace amounts of β-O-4′ and β-β′ linkages. As with the reference reaction the formation of an aldehyde with a ^13^C NMR peak at 191.5 ppm was observed in all samples. This is proposed to form by oxidation of the terminal C3 chain under the oxidative polymerisation conditions. As well as this, molecular weight analysis of the material formed in solution was carried out using analytical ultracentrifugation (AUC), giving a weight average molecular weight of 0.7 kDa for **IE1**, 0.4 kDa for **IE2** and 0.9 kDa for **IE4**, Fig. 4, showing that it was only low molecular weight oligomers that formed even at 38 °C. Figure 4 also shows that **IE1**, **IE2** and **IE4** all appear to be near-monodisperse.

**Figure 5:**
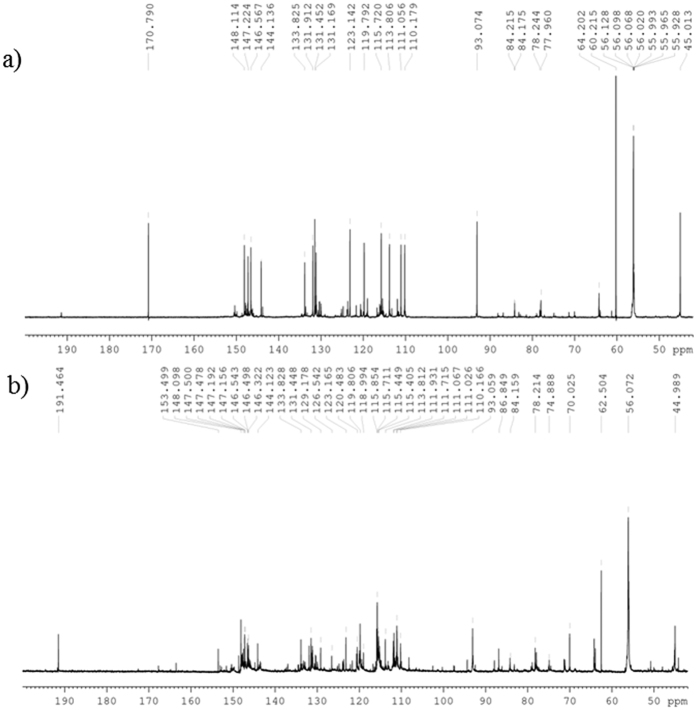
^13^C NMR spectra of (**a**) **IE3** (isoeugenol polymerised at room temperature) (**b**) **IE4** (isoeugenol polymerised at 38 °C. An increasing number of peaks around 84.2 ppm show the presence of significant quantities of the β-O-4′ moiety. Also the increased intensity of the peak at 191.5 ppm with relation to the aromatic peaks shows that an increase in oxidation has occurred promoting the formation of an aldehyde.

Comparison of the ^13^C NMR spectra of **IE1** (Fig. 3b) and **IE3** (Fig. 5) interestingly showed little difference in the proportions of bond types formed between monomers and the oligomer chain, suggesting that reaction time was not a significant factor in the overall structure of the oligomers. However, when comparing the ^13^C NMR spectra of **IE3** and **IE4**, significant differences can be seen, Fig. 5. A significantly larger proportion of the β-O-4′ and β-β′ moieties can be seen in **IE4**, suggesting that the increase in temperature to 38 °C changes the rate at which the different interconnecting moieties form. Additionally, there was a significant increase in the amount of oxidation taking place at 38 °C, as shown by the increase in the ratio between the peak at 191.5 ppm, an aldehyde group, and the aromatic peaks. Despite the differences in the chemical structure, the AUC showed only small differences in the (weight average) molecular weight M_w_. This suggests that polymerisation occurs within the first two days and that further reaction time is not necessary.

This was expected, as visible changes to the reaction mixture were mainly seen during the first two hours. Two hours after the addition of HRP and H_2_O_2_, the solution began to turn cloudy and a yellow oil appeared to be forming which separated from the bulk solution and collected at the bottom of the flask. This continued over the first 24 hours. It is most likely due to the monomer (isoeugenol) becoming less soluble in the presence of greater amounts of water, added with the hydrogen peroxide (3% in water). However, over time it converted from a yellow oil to a brown powder, suggesting that it reacted to form dimers and trimers which are insoluble in water and ethanol mixtures and do not react further. Therefore, any polymerised material found inside the wood would have to have been formed there rather than diffusing into the wood from the bulk solution.

During the polymerisation no colour change was observed in solution apart from the formation of the oil, which was pale yellow, suggesting that no extraction was taking place from the wood. It is possible for ethanol to harvest the extractives and degraded cell wall polymers from highly degraded archaeological wood; however, this is accompanied with a darkening of the solution. The wood used in this study was in a medium state of degradation and the treatment solution contained only 50% v/v ethanol, which is proposed to be non-damaging to the wood under these conditions and impregnation times.

**Figure 6:**
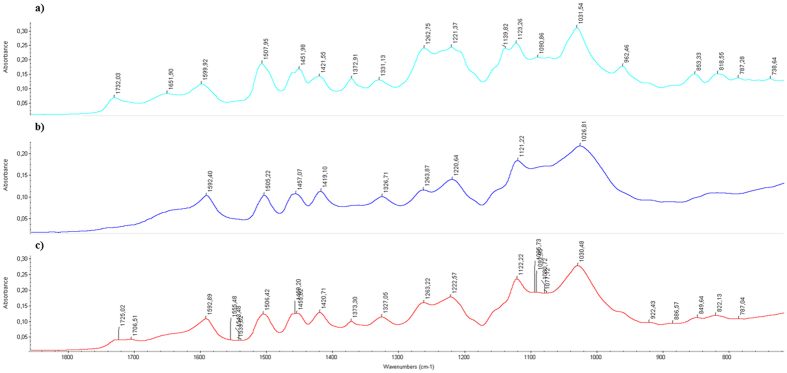
ATR-FTIR spectra of (**a**) polymerised isoeugenol from **IE1**, (**b**) untreated archaeological wood and (**c**) the centre from the treated wood piece of **IE1**. The emergence of a band at 1732 cm^−1^ can be seen in **c** showing the presence of isoeugenol oligomers as it is also seen in spectra **a** but not in **b**.

Following the analysis of the polymerisation products from the solution it was necessary to analyse the wood. This was carried out by FTIR spectroscopy of both the surface and centre of each sample of wood. As the polymerised isoeugenol had a very similar FTIR spectrum to the archaeological wood, indication of its presence *in situ* could only be determined by two bands at 1732 cm^−1^ and 1373 cm^−1^. The band at 1732 cm^−1^ is not present in isoeugenol and is assigned to an aldehyde group on the terminal chain of the oligomer, as can be seen in Fig. 6. The band at 1373 cm^−1^ was present in the polymerised material and treated wood but not the untreated wood or the monomer. This band is proposed to be due to a CH deformation vibration on the lignin-like oligomer^36^. These bands were enough to suggest that polymerised material was present both at the surface and in the core of all four samples (Supplementary Information). Further small changes can be seen in the intensities of some of the bands present; however, it is not known if these are due to the presence of polymerised material or due to slight differences in the cell wall composition as a result of the inhomogeneity of wood.

**Figure 7:**
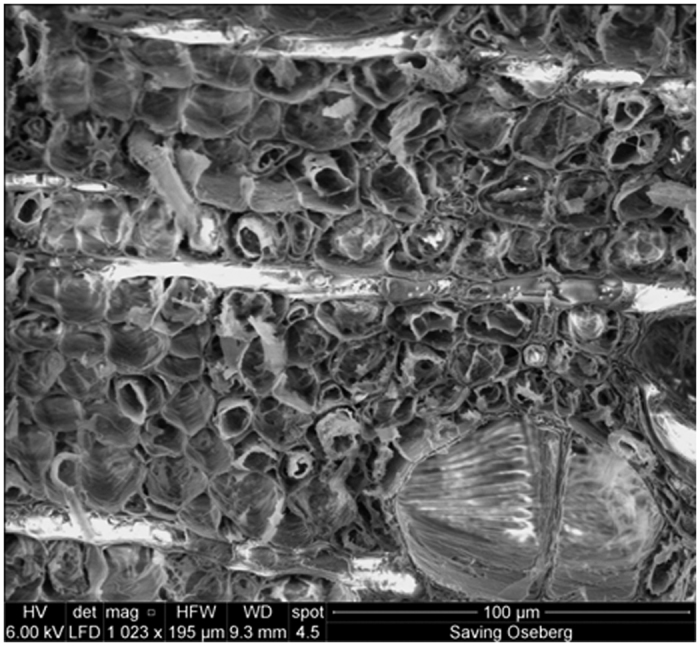
SEM image of the centre of wood sample IE1. The image shows that the wood structure remains open and that **IE1** is not filling the pores of the wood, meaning it may be possible to retreat the sample if needed in the future.

Following analysis by FTIR spectroscopy, cross-sections from the centre of all samples were taken and visualised by scanning electron microscopy (SEM). All samples had an open wooden structure, as expected for freeze-dried archaeological wood. However, despite the presence of lignin-like oligomers, indicated by FTIR spectroscopy, no material could be seen in the SEM images, Fig. 7 (all SEM images can be found in Supplementary Information). This suggests that the oligomers formed are coating the cell surfaces in a thin layer rather than filling the pores. This could be useful, as it will allow for future re-treatments to be carried out if needed without the removal of the existing lignin-like consolidant.

Despite a relatively large proportion of the formed lignin-like oligomers remaining in solution, these may have a use beyond *in situ* polymerisation. A previous study by our group has found that lignin-like oligomers can offer consolidation to waterlogged archaeological wood. Any remaining material after *in situ* polymerisation could be used for other treatments of waterlogged archaeological wood, as described in this group’s previous papers^26^,^29^. As such, this method can be seen to produce minimal waste as even the ‘waste product’ may have a use within the field of conservation.

## Conclusion

Our study shows that isoeugenol can be polymerised *in situ* in waterlogged archaeological wood with HRP giving a structure rich in the chemically stable β-5′ moiety, with limited amounts of the less chemically stable β-O-4′ moiety. The consolidation effect of the *in situ* polymerisation was not investigated at this time as it was necessary to first establish that *in situ* polymerisation could occur, although it was noted that all of the treated samples were stable enough to be handled and to have cross-sections cut for analysis. It is proposed that a follow up investigation looking at relative concentrations and addition rates of isoeugenol, HRP and H_2_O_2_ and the consolidation abilities should be carried out. Given that the polymerisation occurs *in situ* and that the waste material has a potential re-use, this method shows promise for development into a green consolidation method for waterlogged archaeological wood.

## Materials and Methods

All chemicals and reagents were purchased through Sigma Aldrich Norway. HRP type II essentially salt-free, lyophilized powder (150–250 units/mg) was used for all reactions. Waterlogged archaeological wood samples, excavated in 2005 from Slagen Presterød, Vestfold County, Norway were kindly donated by the Museum of Cultural History, University of Oslo. The wood genus was identified by light microscopy as alder (*Alnus* spp.). Samples used for *in situ* polymerisation were cut from slices with a diameter of 10 cm and longitudinal thickness of 1 cm still in waterlogged condition. Each sample had an approximate volume of 1 cm^3^ and rough dimensions of 2 × 1 × 0.5 cm. The average density of both the core and outer regions was 0.146 g/mL and the maximum moisture content was 620%.

### Polymerisation of isoeugenol

Isoeugenol (0.5 mL, 3.3 mmol) was added to a solution of water and ethanol (50 mL, 4:6) and the mixture was heated to 38 °C. The pH of the solution was measured and a solution of sodium hydrogen carbonate (0.01 M) was added to raise the pH to 6. Following this HRP (2.5 mg, approximately 450 units) and H_2_O_2_ (7.5 mL, 3.3 mmol, 3% in water) were added and the mixture was stirred at 38 °C in a sealed reaction flask for 1.5 h before cooling. The product was then extracted with ethyl acetate (2 × 100 mL), the organic layers were combined and washed with sat. aq. NaCl (3 × 50 mL) and dried over MgSO_4_. The solvent was removed under reduced pressure to give a brown oil.

### General Procedure for *in situ* polymerisation of isoeugenol in archaeological wood

The wood sample was cut into small chunks of approximately 1–2 cm^3^. A wood piece (*ca.* 1–2 cm^3^) was placed directly into a solution of isoeugenol (0.15 mL, 1.00 mmol) in water and ethanol (10 mL, 1:1) for 2–7 days at either room temperature or 38 °C in a sealed reaction flask to allow impregnation of the monomer. Following this HRP (0.66 mg, approximately 100 units) and H_2_O_2_ (2.3 mL, 1.0 mmol, 3% in water) was added followed by gentle shaking. The solution was allowed to react with gentle shaking multiple times a day for 2–15 days. Upon completion of the polymerisation, as determined by the loss of isoeugenol in solution by TLC (2:1 petroleum ether-ethyl acetate), the wood sample was removed from the reaction mixture and placed in water for 6 h to remove ethanol prior to freeze drying. The remaining solution was extracted with ethyl acetate (3 × 15 mL), washed with sat. aq. NaCl (3 × 15 mL) and dried over MgSO_4_ before being concentrated *in vacuo*.

### NMR spectroscopy of DHPs

NMR spectra were recorded on a Bruker Avance II 600 MHz Spectrometer equipped with a TCI cryo probe and a “SampleCase”, using DMSO-*d*_*6*_ as solvent. The sample of the DHP was dissolved in DMSO-*d*_*6*_ at 55–100 mg/mL depending on sample amount and solubility. NMR spectra were acquired and processed using Topspin 3.2 pl6 and ^13^C NMR spectra were recorded using a z-restored spin-echo pulse program to achieve a straight baseline free of hump, dip or roll^37^. An acquisition time of 0.9 s, relaxation delay of 10 s, a dead time of 70 μs, 5120 scans and a spectral width of 239 ppm at 300 K was used for all acquisitions. Assignment of main lignin-like features of DHPs was performed according to Ralph *et al*^38^.

### Analytical Ultracentrifugation

Polymerised material was assayed for molar mass using a Beckman Optima XL-I Analytical Ultracentrifuge (AUC). Sedimentation equilibrium profiles were recorded using absorbance optics at a wavelength of 360 nm, at a rotor speed of 50 k RPM (~195 000 g) and temperature of (20.0 ± 0.1)°C. 12 mm path length double sector cells were employed with titanium centrepieces, sapphire windows and aluminium housings. Cells contained 100 μL polymerised material at 1.0 mg/mL dissolved in 100% DMSO (non-deuterated) in the solution channels of each cell, and DMSO in the reference solvent channels. Samples were centrifuged until equilibrium was achieved (approximately 24 h).

Data were analysed using SEDFIT-MSTAR v1^39^. Apparent, weight-average, molar masses (M_w,app_) were determined by extrapolating an operational point average molar mass known as the M*(r) function to the cell base r = b, based on the identity M*(b) = M_w,app_^40^. Polydispersity of material was assessed from the distribution of local or point weight average molar masses M_w,app_(r) with radius r. M_w,app_(r) are calculated from the local slopes along the ln(c) vs r^2^ plots^39^. M_w,app_ can be approximated as M_w_, the ideal weight average molar mass, and M_w,app_ can be approximated as M_w_ since thermodynamic non-ideality will be negligible for such small molecules at low concentration. A partial specific volume of 0.61 mL/g^41^,^42^ and density of DMSO (1.10 g/mL) were used.

### ATR-FTIR spectroscopy

Attenuated Total Reflectance Fourier Transform Infra-Red (ATR-FTIR) spectroscopic measurements were performed on a Thermo Scientific iS50 FTIR Spectrometer. Slices of treated wood were taken using a micro blade razor from both the surface and centre of each piece. These were then subjected to ATR-FTIR spectroscopy by placing directly onto the crystal using 32 scans at resolution 4 cm^−1^ and spectral range: 4000–400 cm^−1^.

### SEM imaging

Scanning Electron Microscope (SEM) images were recorded on an FEI Quanta 450 Scanning Electron Microscope. Electron images were taken using a large field detector (LFD) under low vacuum (LV) mode in order to avoid charging of the samples. The other parameters (voltage, spot size, pressure, and working distance) were modified depending on the sample with average values of HV 6.0 kV, spot size 4.5, pressure 100 Pa and working distance of 7.9 mm.

## Additional Information

**How to cite this article:** McHale, E. *et al. In situ* polymerisation of isoeugenol as a green consolidation method for waterlogged archaeological wood. *Sci. Rep.* **7**, 46481; doi: 10.1038/srep46481 (2017).

**Publisher's note:** Springer Nature remains neutral with regard to jurisdictional claims in published maps and institutional affiliations.

## Supplementary Material

Supplementary Information

## Figures and Tables

**Table 1:** Reaction conditions for the *in situ* polymerisation of isoeugenol in wood.

Sample	Impregnation of isoeugenol (days)	Reaction time (days)	Temperature (°C)	M_w_ (kDa) of collected material	Presence of FTIR band at 1730 cm^−1^ *in situ*
IE1	2	2	r.t	0.7	Y
IE2	4	4	r.t	0.4	Y
IE3	7	15	r.t	—	Y
IE4	7	15	38	0.9	Y
