# Supplementary material for: In situ polymerisation of isoeugenol as a green consolidation method for waterlogged archaeological wood
Source: Sci Rep. 2017 Apr 27;7:46481. doi: 10.1038/srep46481 (PMC5406834; doi:10.1038/srep46481)
Supplement: Supplementary Information [file srep46481-s1.pdf]

In situ polymerisation of isoeugenol as a green consolidation method for waterlogged archaeological wood – Supplementary Information

Emily McHale*,[a,b] Calin C. Steindal,[a] Hartmut Kutzke,[a] Tore Benneche[b] and Stephen E. Harding* [c]

[a] Museum of Cultural History, University of Oslo, Postbox 6762 St. Olavs plass, 0130 Oslo, Norway
[b] Department of Chemistry, University of Oslo, Postbox 1033 Blindern, 0315 Oslo, Norway
[c] National Centre for Macromolecular Hydrodynamics, University of Nottingham, Sutton Bonington, LE12 5RD, United Kingdom

*Corresponding authors: email: emily.mchale@khm.uio.no, steve.harding@nottingham.ac.uk

ATR-FTIR spectra for all samples: Figs. S1-S3

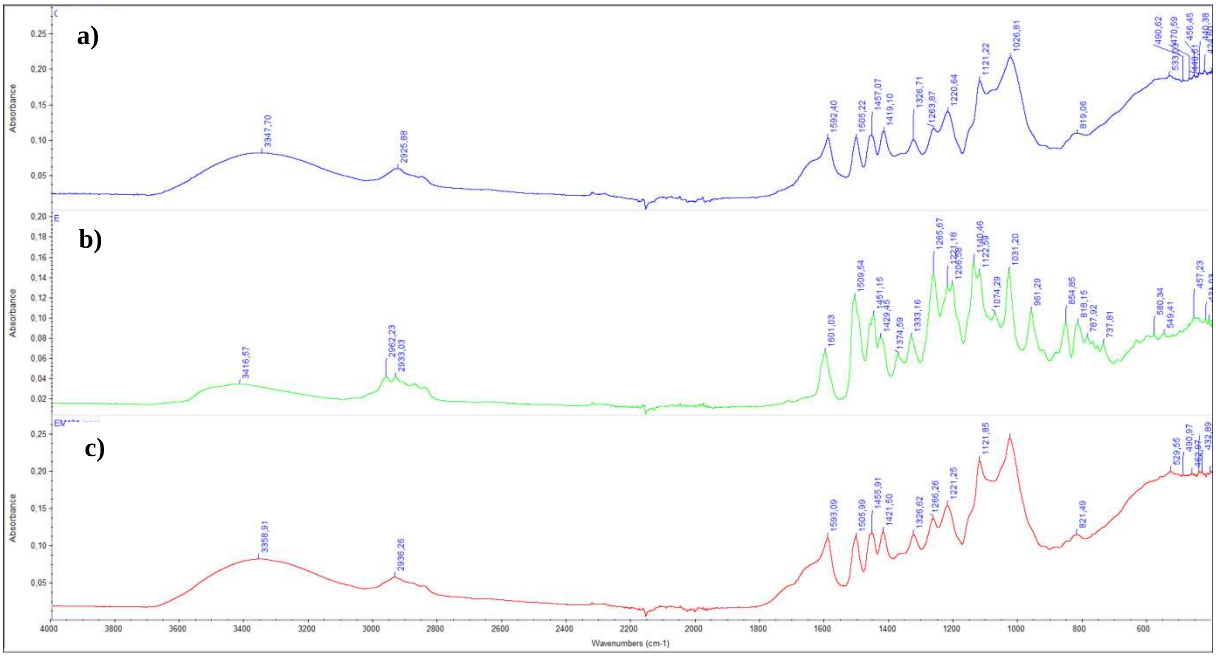

Figure S1: ATR-FTIR spectra of **IE2** with **a)** untreated reference, **b)** polymerised material, **c)** centre of treated sample. The emergence of a band at 1725 cm^{-1} can be seen in **c** showing the presence of **IE2** in the centre of the wood.

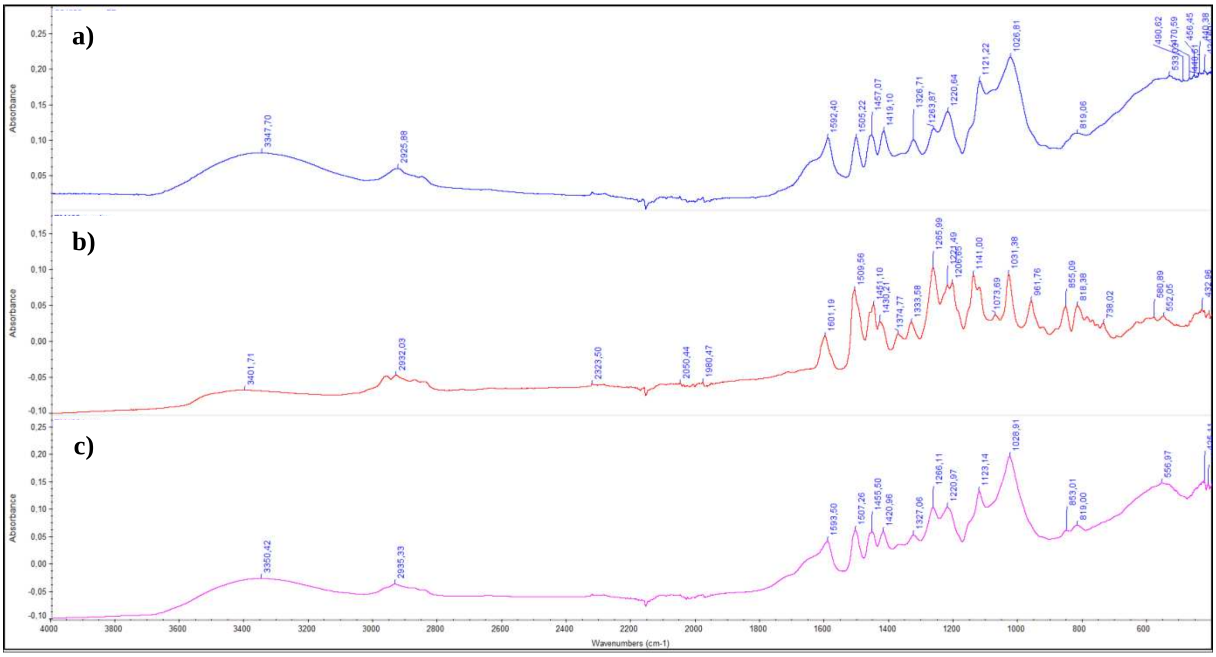

Figure S2: ATR-FTIR of **IE3** with **a)** untreated reference, **b)** polymerised material, **c)** centre of treated sample. The emergence of a band at 1725 cm^{-1} can be seen in **c** showing the presence of **IE3** in the centre of the wood.

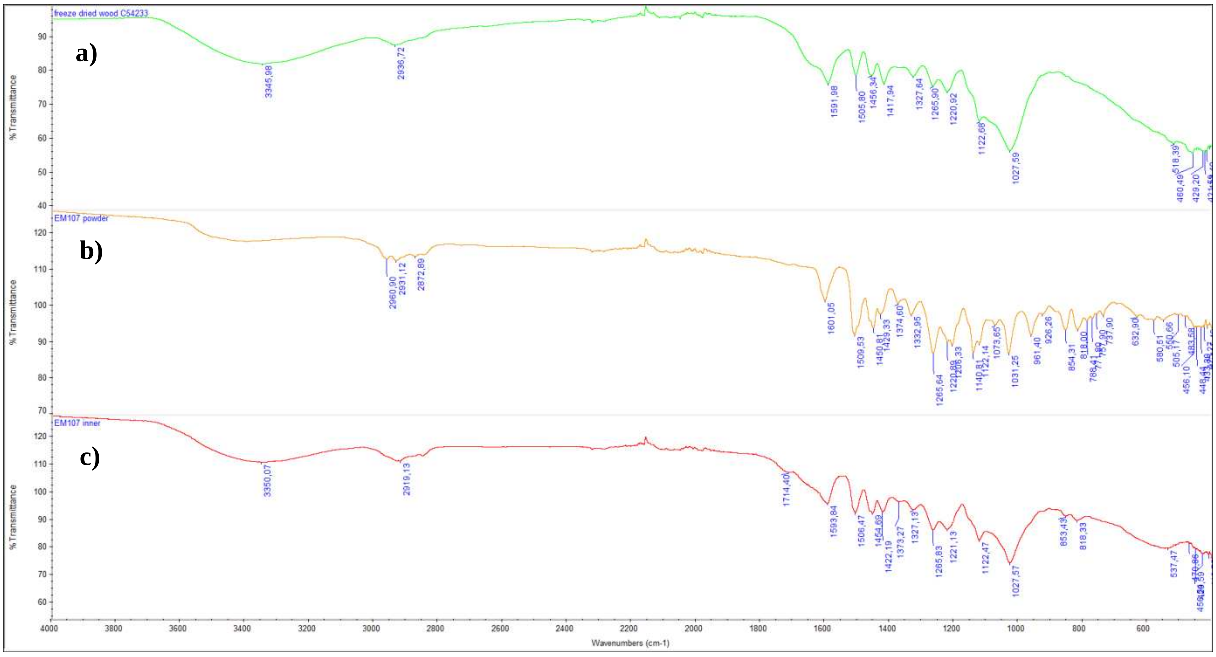

Figure S3: ATR-FTIR of **IE4** with **a)** untreated reference, **b)** polymerised material, **c)** centre of treated sample. The emergence of a band at 1725 cm^{-1} can be seen in **c** showing the presence of **IE3** in the centre of the wood.

SEM images of cross-sections of all samples (treated and untreated): Figs. S4-S6

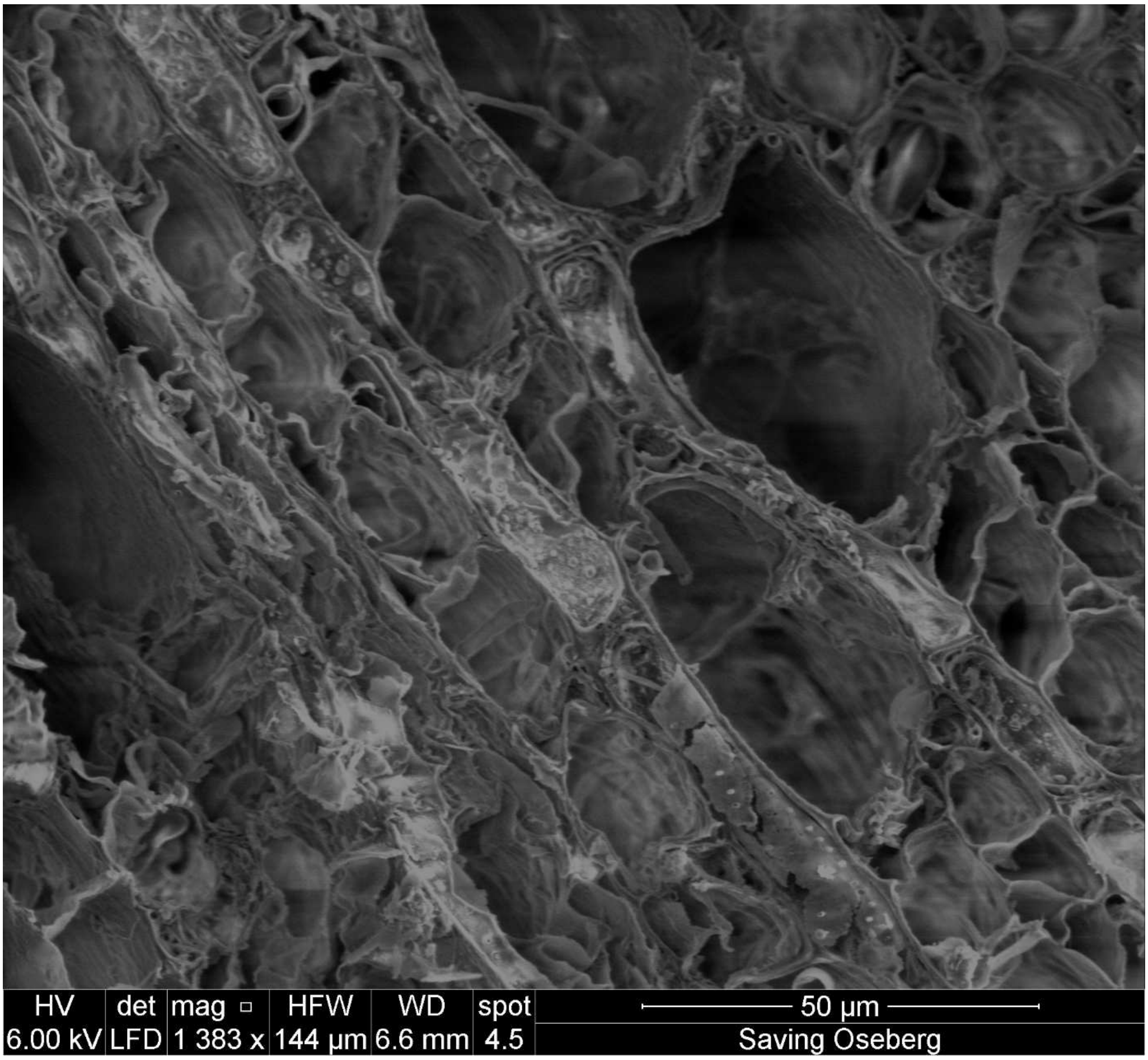

Figure S4: SEM image of a cross-section of **IE2** after treatment. The image shows that the wood structure remains open and that **IE2** is not filling the pores of the wood meaning it would be possible to retreat the sample if needed in the future.

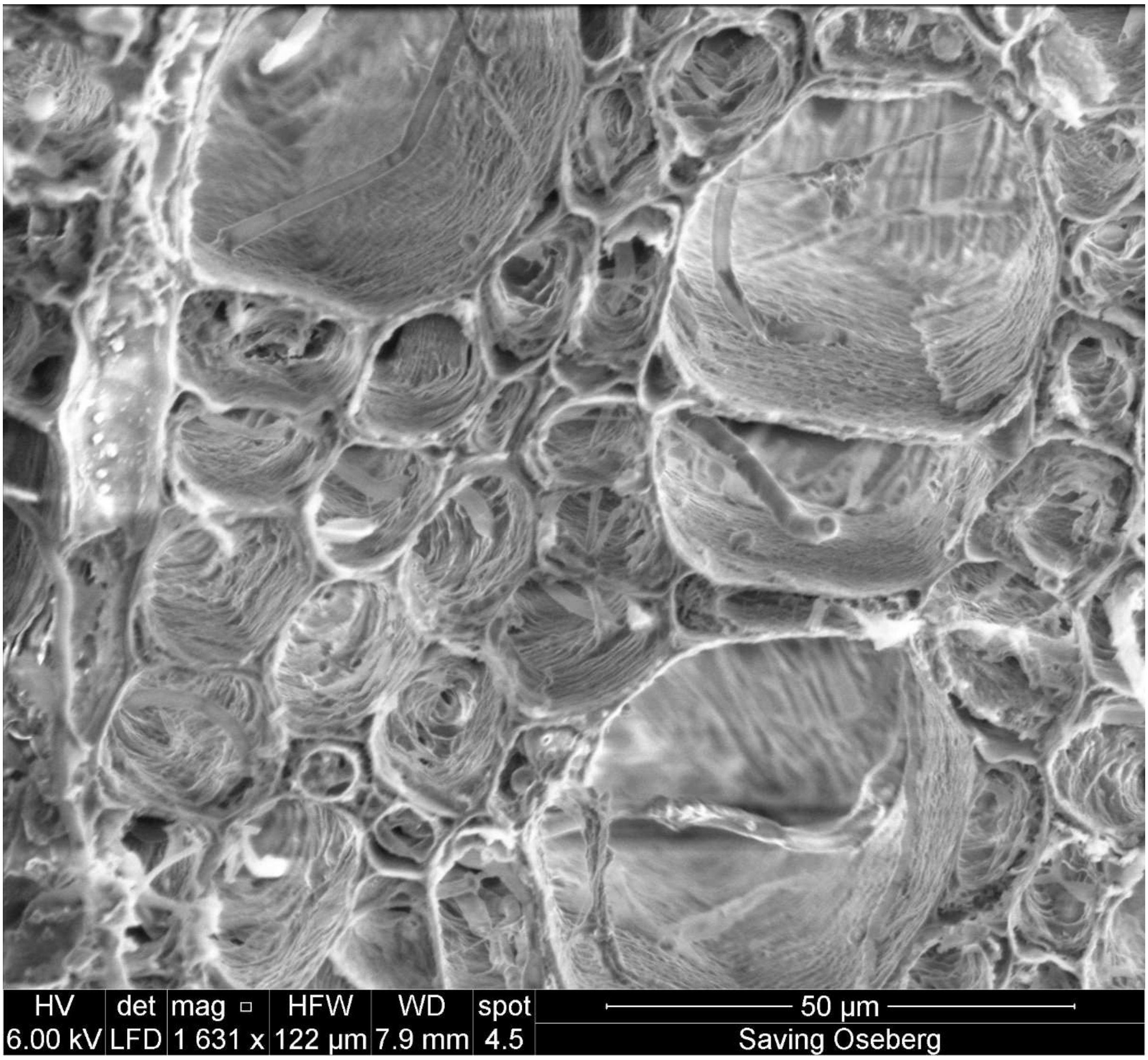

Figure S5: SEM image of a cross-section of **IE3** after treatment. The image shows that the wood structure remains open and that **IE3** is not filling the pores of the wood meaning it would be possible to retreat the sample if needed in the future.

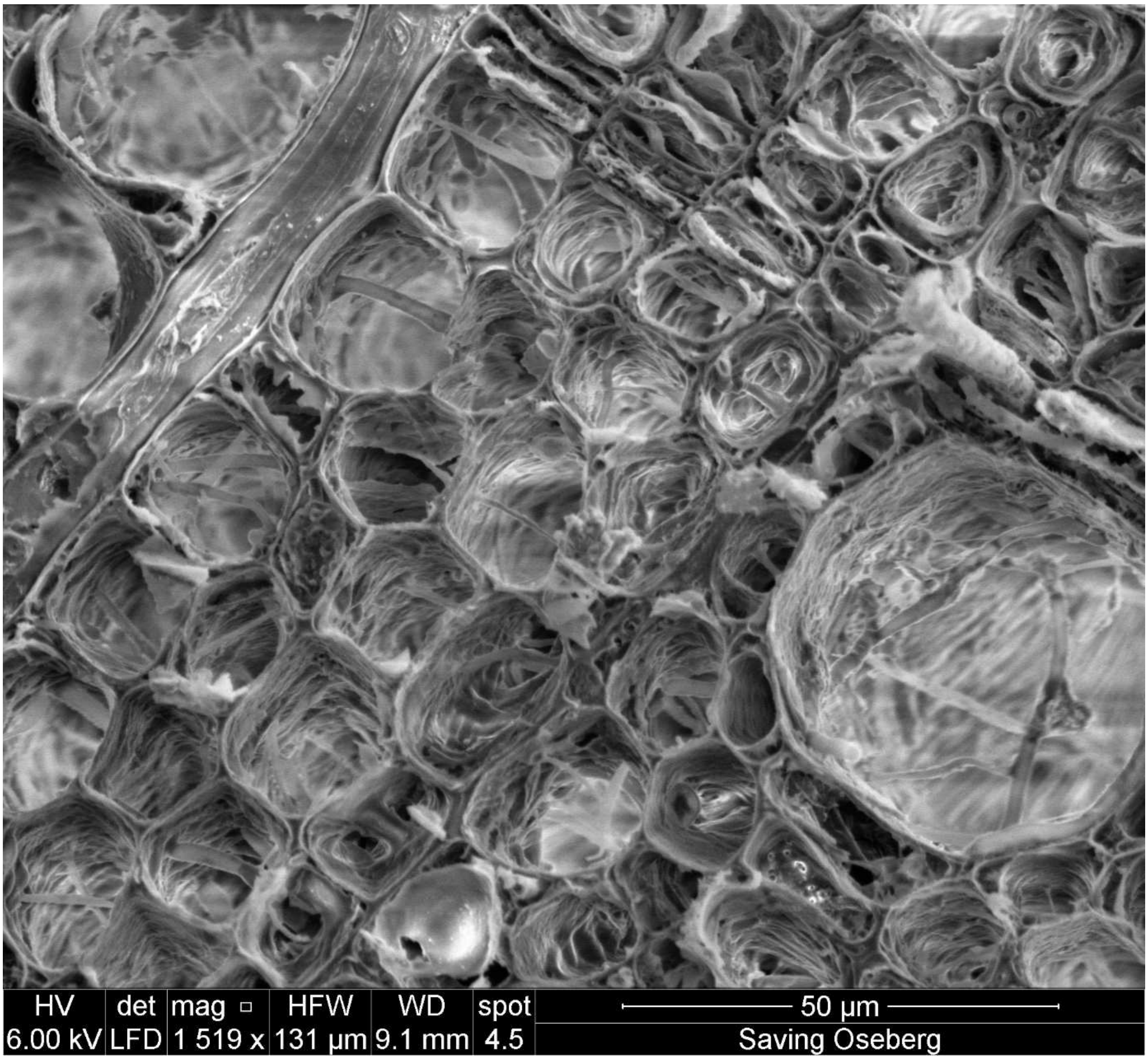

Figure S6: SEM image of a cross-section of **IE4** after treatment. The image shows that the wood structure remains open and that **IE4** is not filling the pores of the wood meaning it would be possible to retreat the sample if needed in the future.
